# Successful Diagnosis by Video-Assisted Thoracoscopic Surgical Lung Biopsy in a Case of Progressive Primary Pulmonary Extranodal Natural Killer/T-cell Lymphoma, Nasal Type: A Case Report

**DOI:** 10.7759/cureus.34227

**Published:** 2023-01-26

**Authors:** Satoshi Anai, Hiroki Gushiken, Shigeki Chinen, Akira Gakiya, Masaya Kiyuna

**Affiliations:** 1 Division of Respiratory Medicine, Yuuai Medical Center, Tomigusuku City, JPN; 2 Division of Thoracic Surgery, Yuuai Medical Center, Tomigusuku City, JPN; 3 Division of Pathology, Yuuai Medical Center, Tomigusuku City, JPN

**Keywords:** video-assisted thoracoscopic surgery, case report, primary pulmonary extranodal natural killer/t-cell lymphoma nasal type, flow cytometry, diffuse ground-glass opacities

## Abstract

Malignant pulmonary lymphoma is very rare and the majority of which are B-cell lymphomas. Since primary pulmonary extranodal natural killer (NK)/T-cell lymphoma, nasal type (ENKL) is difficult to diagnose and associated with poor prognosis and aggressive course, some cases are diagnosed at the postmortem autopsy. We report a case of a 53-year-old man with primary pulmonary ENKL diagnosed by video-assisted thoracoscopic surgery (VATS) lung biopsy. This case explains the importance of VATS lung biopsy and in-depth evaluation, including flow cytometry, chromosome, genetic, and immunostaining tests, when primary pulmonary malignant lymphoma is suspected.

## Introduction

Primary pulmonary malignant lymphoma is defined as lymphoma confined to the lung with or without hilar lymph node metastasis at diagnosis or for up to three months thereafter [1]. Primary non-Hodgkin's lymphoma of the lung is a very rare disease, accounting for approximately <1.0% of all non-Hodgkin's lymphomas [2]. Primary non-Hodgkin's malignant lymphoma of the lung accounts for approximately 0.3% of all lung tumors [2]. Furthermore, the majority of primary non-Hodgkin's lymphomas of the lung are B-cell lymphoma, while extranodal natural killer (NK)/T-cell lymphoma, nasal type (ENKL) account for 7.4% (2/27) [1]. Furthermore, not only are there few case reports on primary pulmonary ENKL but there are also no coherent reports of sufficient numbers of cases. Unfortunately, ENKL is difficult to diagnose and is mostly a postmortem diagnosis with an extremely poor prognosis. In this report, we describe a case of primary pulmonary ENKL, successfully diagnosed by video-assisted thoracoscopic surgery (VATS) lung biopsy.

## Case presentation

**Figure 1:**
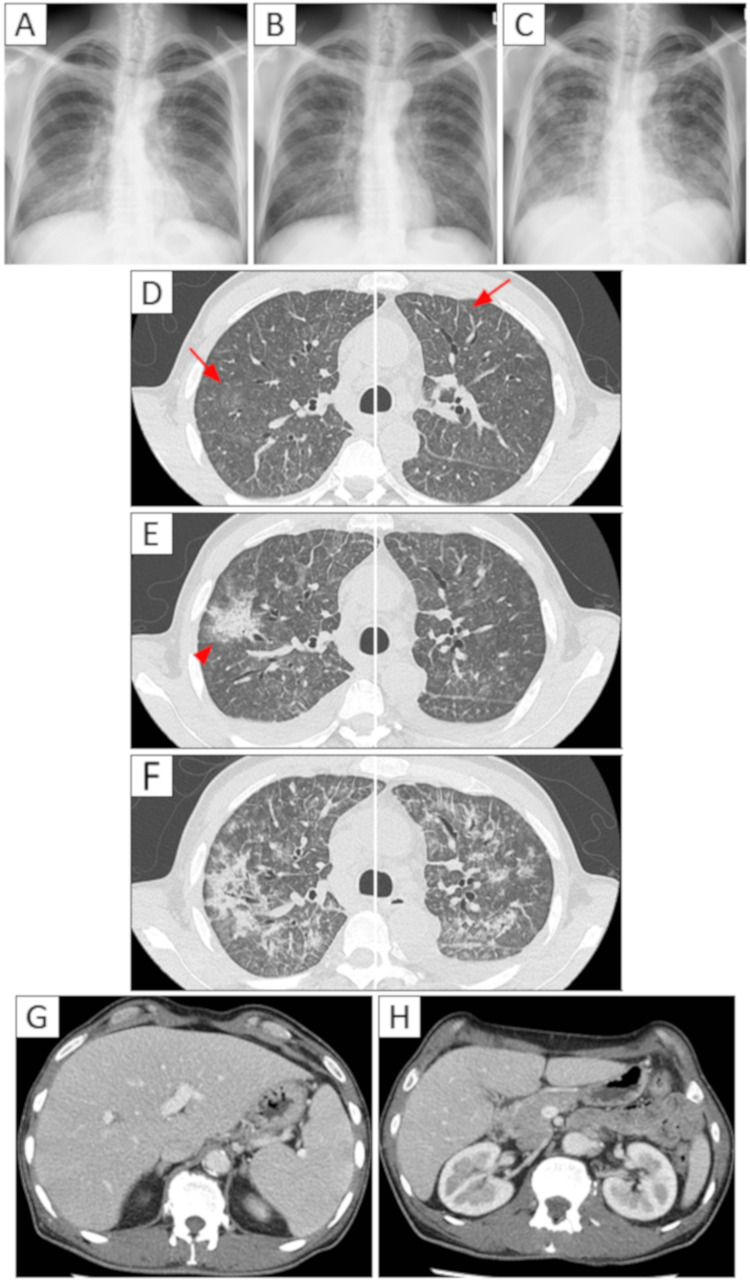
CXR and chest CT images on admission and after admission. CXR on admission showed diffuse ground-glass opacities in both lungs (A). After discharge from the hospital, CXR showed new infiltrative shadows in addition to diffuse ground-glass opacities in both lungs (B). Chest CT on admission showed diffuse ground-glass opacities in both lungs with thickening of the interlobular septal wall (red arrow) (D). The chest CT showed new infiltrative shadows in addition to diffuse ground-glass opacities in both lungs (red arrowhead) (E). CXR and chest CT before VATS lung biopsy showed further exacerbation of the previous imaging findings (C, F). Contrast-enhanced CT of the abdomen on admission, showing hepatosplenomegaly (G) and bilateral kidney enlargement (H).

A 53-year-old man was admitted to our hospital with 15 kg weight loss and anorexia over 6 months period. On admission, physical examination revealed clear consciousness, a temperature of 36.4°C, blood pressure of 104/66 mmHg, pulse rate of 92 beats/min, respiratory rate of 24/min, and oxygen saturation of 97% (room air). Chest auscultation revealed slight end-inspiratory fine crackles in both lungs. The heart sounds were normal. Chest radiography (CXR) revealed ground-glass opacities in both lung fields (Figure 1A). Chest computed tomography (CT) revealed diffuse ground-glass opacities with interlobular septal thickening (Figure 1D). The mediastinal lymph nodes were mildly enlarged. Mild hepatosplenomegaly and bilateral kidney enlargement were also observed (Figures 1G, 1H). Inflammatory markers were elevated as shown in Table 1. Hyponatremia and hypokalemia were also observed (Table 1). We considered sarcoidosis, lymphangitic carcinomatosis owing to gastric cancer, malignant lymphoma, lymphoproliferative disease, or anti-neutrophil cytoplasmic antibody (ANCA) - associated vasculitis as possible differential diseases. Tests performed to differentiate these diseases showed mildly elevated lactate dehydrogenase (LDH), soluble interleukin-2 receptor (sIL-2R), and angiotensin-converting enzyme (ACE) levels were observed (Table 1).

**Table 1:** Laboratory data on admission WBC: White blood cell, Hb: Hemoglobin, Plt: Platelet, CRP: C-reactive protein, ANA: Anti-nuclear antibody, RF: Rheumatoid factor, PR3-ANCA: Proteinase-3-antineutrophil cytoplasmic antibody, MPO-ANCA: Myeloperoxidase anti-neutrophil cytoplasmic antibody, CEA: Carcinoembryonic antigen, CYFRA: Cytokeratin 19 fragment, ProGRP: Progastrin-releasing peptide, sIL-2R: soluble interleukin-2 receptor, COVID-19: coronavirus disease 2019, PCR: polymerase chain reaction, CMV: Cytomegalovirus, IGRA: Interferon-gamma release assay, HTLV-1: Human T-cell leukemia virus type 1, TP: Total protein, Alb: Albumin, AST: Aspartate aminotransferase, ALT: Alanine aminotransferase, LDH: Lactate dehydrogenase, BUN: Blood urea nitrogen, Cre: Creatinine, UA: Na: Serum sodium concentration, Cl: Serum chloride concentration, K: Serum potassium concentration, KL-6: Sialylated carbohydrate antigen KL-6, SPD: Surfactant protein D, ACE: Angiotensin-converting enzyme, AVP: Arginine vasopressin, TSH: Thyroid-stimulating hormone, FT4: Free thyroxine, CD4: Cluster of differentiation 4, CD8: Cluster of differentiation 8

Test	Result	Reference value	Units
Peripheral blood			
WBC	5000	3300 - 8600	/μl
Neut	66.8	38.5 - 80.5	%
Lymph	24.4	16.5 - 49.5	%
Mono	7.4	2.0 - 10.0	%
Eosino	0.4	0.0 - 8.5	%
Baso	1.0	0.0 - 2.5	%
Hb	12.3	13.7 - 16.8	g/dL
Plt	30.0 x 10^4^	15.8 - 34.8	/mL
Serology			
CRP	1.29	0.00 - 0.14	mg/dl
ANA	<1:40	<1:40	
RF	<8	<15	IU/mL
PR3-ANCA	1.0	<3.5	U/mL
MPO-ANCA	1.0	<3.5	U/mL
Tumor marker			
CEA	2.2	<5.0	ng/mL
CYFRA	2.3	0 - 3.5	ng/mL
ProGRP	61.1	<81.0	pg/mL
sIL-2R	1770	157 - 474	U/mL
Urinalysis			
Protein	4+	-	
Sugar	-	-	
Occult blood	-	-	
Urinary osmolality	732	50 - 1300	mOsm/kgH_2_O
Urinary Na	46.8	40 - 90	mEq/L
Infection			
Pneumococcal urinary antigen test	negative	negative	
Legionella urinary antigen test	negative	negative	
COVID-19 PCR test	negative	negative	
β-d-glucan	16.6	<20	pg/mL
CMV antigenemia assays	negative	negative	
IGRA	negative	negative	
HTLV-1 Antibody	negative	negative	
Biochemistry			
TP	6.7	6.6 - 8.1	g/dL
Alb	2.8	4.1 - 5.1	g/dL
AST	62	13 - 30	IU/L
ALT	44	10 - 42	IU/L
LDH	451	124 - 222	IU/L
BUN	14	8 - 20	mg/dL
Cre	0.93	0.65 - 1.07	mg/dL
UA	4.0	3.7 - 7.8	mg/dL
Na	124	138 -145	mmol/L
Cl	85	101 - 108	mmol/L
K	2.9	3.6 - 4.8	mmol/L
KL-6	567	<500	u/mL
SPD	22.7	<110	ng/mL
ACE	27.4	8.3 - 21.4	IU/L
Serum osmolality	255	275 - 290	mOsm/kgH_2_O
AVP	0.5	<3.8	pg/mL
Plasma renin activity	3.2	0.2 - 2.7	ng/ml/h
Cortisol	9.4	3.7 - 19.4	μg/dL
TSH	1.34	0.61 - 4.23	μIU/mL
FT4	0.97	0.70 - 1.48	ng/dL
BALF			
Rt B3 Fluid recovery Rate	73.3		%
Cell count	304 x 10^4^	3.1 x 10^4^ - 35.1 x 10^4^	/ml
Neut	1	0 - 3.1	%
Lymph	41	1.9 - 34.3	%
Mφ	58	54.6 - 97.1	%
Eosino	0	0 - 1.1	%
CD4/CD8 ratio	0.28	1.0 - 2.0	
Arterial blood gase analysis (Room air)			
pH	7.52	7.35 - 7.45	
PCO_2_	34	35 - 45	mmHg
PO_2_	63	83 - 108	mmHg
HCO_3_	27.8	21.8 - 26.9	mmHg
Pulmonary function test			
%VC	85.6	>80	%
FEV1%	98.8	>70	%
%DLCO	47.6	>80	%

**Figure 2:**
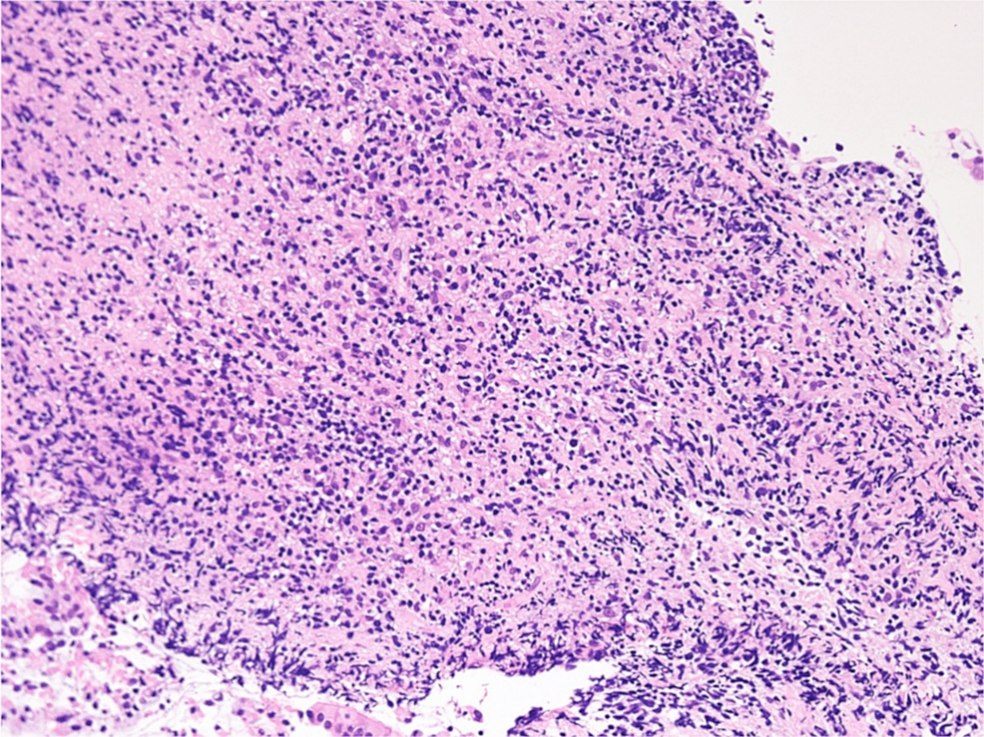
Pulmonary pathology by TBB. Pulmonary pathology by TBB showed infiltration of lymphocytes and plasma cells in the interstitium. Some slightly enlarged lymphocyte-like cells were seen, but it was difficult to distinguish them from lymphoma (C HE staining, original magnification, ×400).

**Figure 3:**
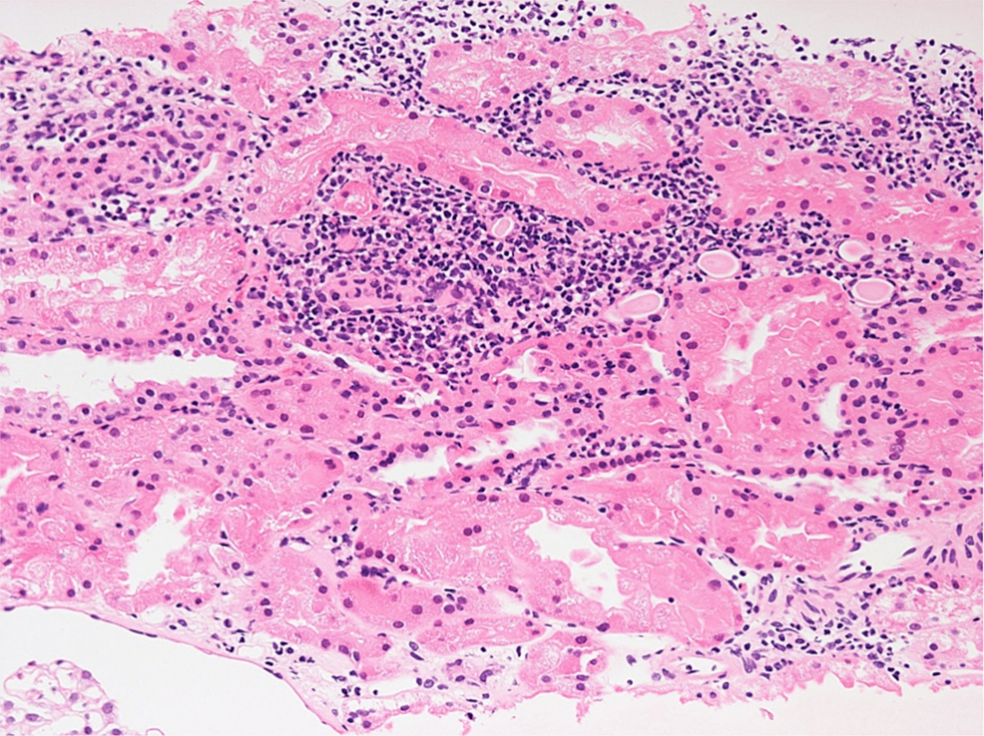
Renal biopsy tissue. Lymphocytic infiltrate was observed in the interstitium (B HE staining, original magnification, ×400).

During the hospital stay, an upper gastrointestinal endoscopy was performed, but no specific findings were observed. Therefore, sarcoidosis and SIADH were suspected. After admission, plasma and urine osmolalities, urine electrolytes, and hormone tests were performed (Table 1). He met the diagnostic criteria and was diagnosed with SIADH. Salt loading, fluid restriction, furosemide, and tolvaptan were administered to treat hyponatremia. Furthermore, the patient underwent bronchoscopy with bronchoalveolar lavage (BAL) in the right middle lobe and transbronchial biopsy (TBB) from the right upper lobe. The recovery rate of BAL was greater than 50%. BAL cytology showed elevated cell counts and lymphocyte count ratios with a decreased cluster of differentiation (CD) 4/CD8 ratio (Table 1). TBB showed lymphocyte infiltration, but no non-caseating epithelioid cell granulomas were seen in sarcoidosis (Figure 2). Gallium scintigraphy, ophthalmologic and cardiovascular examination revealed no findings suggestive of sarcoidosis. Bilateral kidney enlargement and electrolyte abnormalities suggested nephritis as a complication; therefore, a renal biopsy was performed. The pathological diagnosis was interstitial nephritis (Figure 3). With some improvement in symptoms and a strong desire to be discharged, the patient was temporarily discharged 14 days after admission with a directive for a thorough outpatient evaluation.

**Figure 4:**
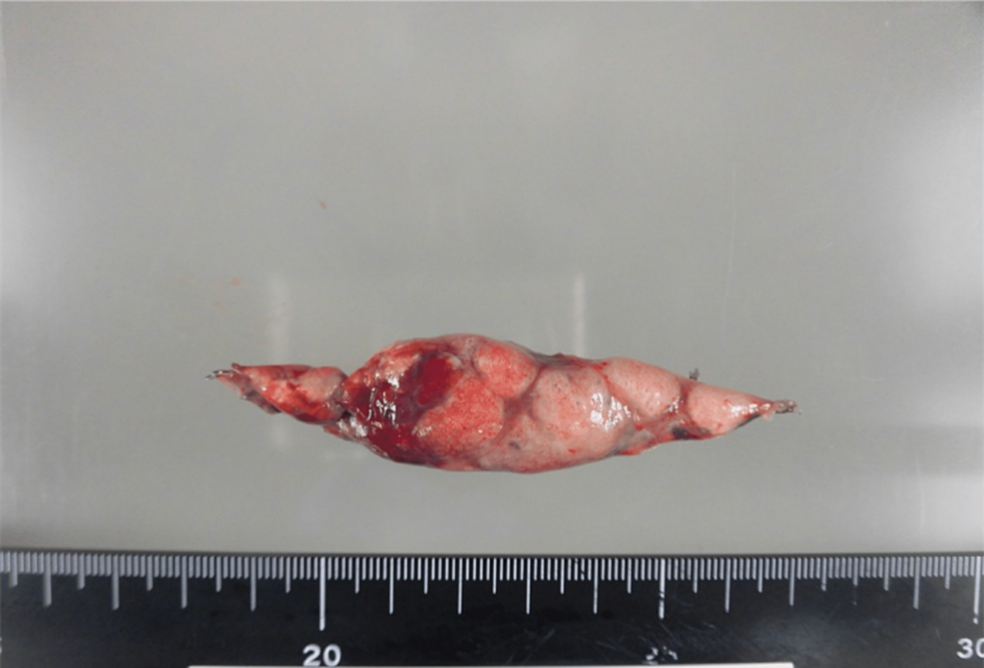
Lung tissue biopsied by VATS.

**Figure 5:**
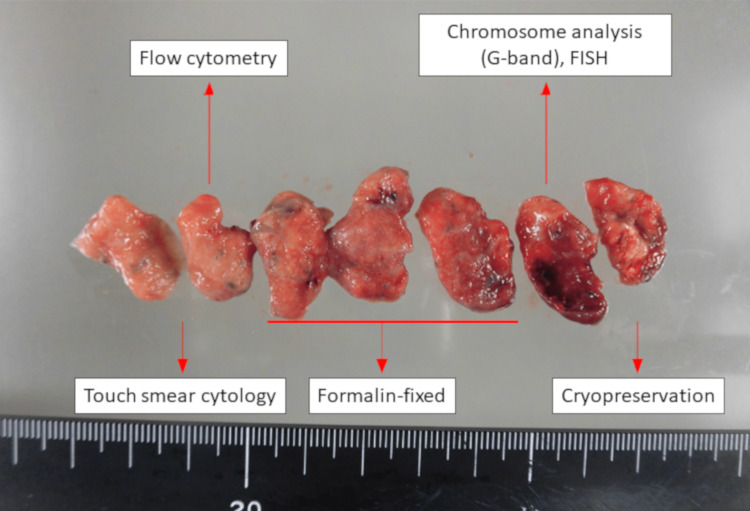
Lung tissue sectioning by a pathologist to differentiate primary pulmonary malignant lymphoma.

However, seven days after discharge, he was readmitted to the hospital for management of decreased appetite, worsening fatigue, and worsening respiratory condition (oxygen saturation 90%, oxygen 3 L/min). The CXR and CT scans, in addition to diffuse ground-glass opacities, also showed the appearance of mass-like infiltrates with moderate pleural effusion in both lungs (Figures 1B, 1E). LDH and sIL-2R levels were mildly elevated (LDH 396 IU/L, sIL-2R 1,900 U/mL). Since LDH and sIL-2R are often elevated in patients with malignant lymphoma, considering the possibility of primary pulmonary malignant lymphoma or lymphoproliferative disease, VATS lung biopsy was performed on the seventh day of re-hospitalization. In VATS lung biopsy, the patient underwent partial resection of the right upper lobe. Subsequently, the biopsy specimen was cut into 1 cm thick slices in a plane perpendicular by the pathologist for flow cytometry, chromosome analysis, genetic testing and immunohistological examination. These examinations were performed using integrated analysis of malignant lymphoma, ML-NET (Figures 4, 5) [3].

**Figure 6:**
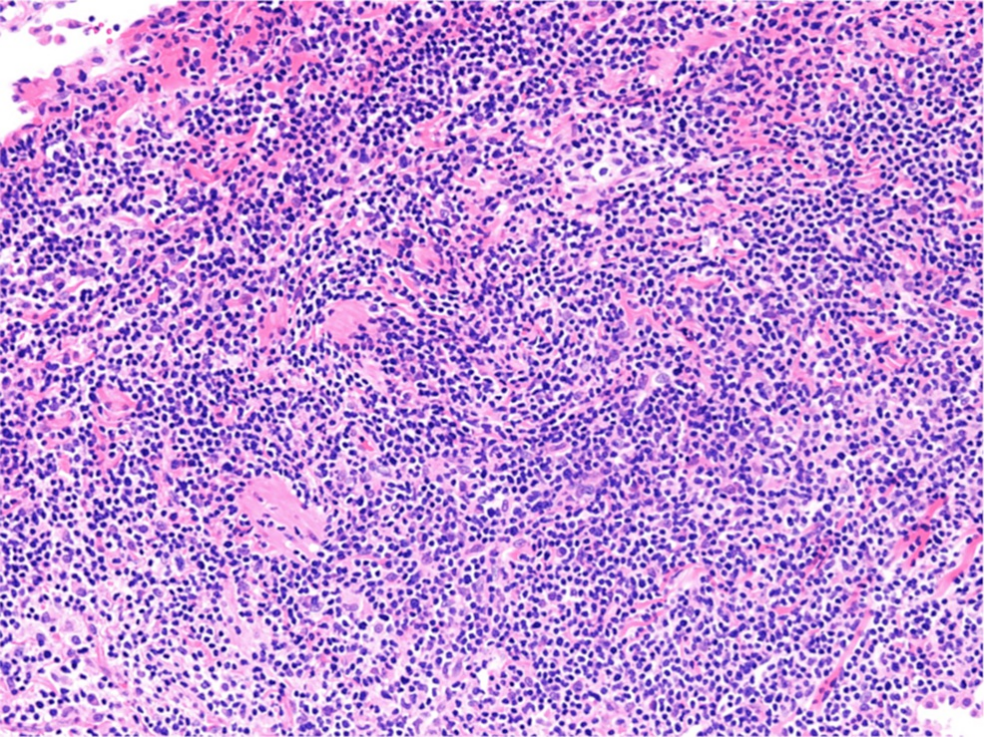
VATS lung biopsy tissue. Infiltration of lymphocytes and plasma cells was observed in the interstitium. Some slightly enlarged lymphocyte-like cells were seen (HE staining, original magnification, ×400).

**Figure 7:**
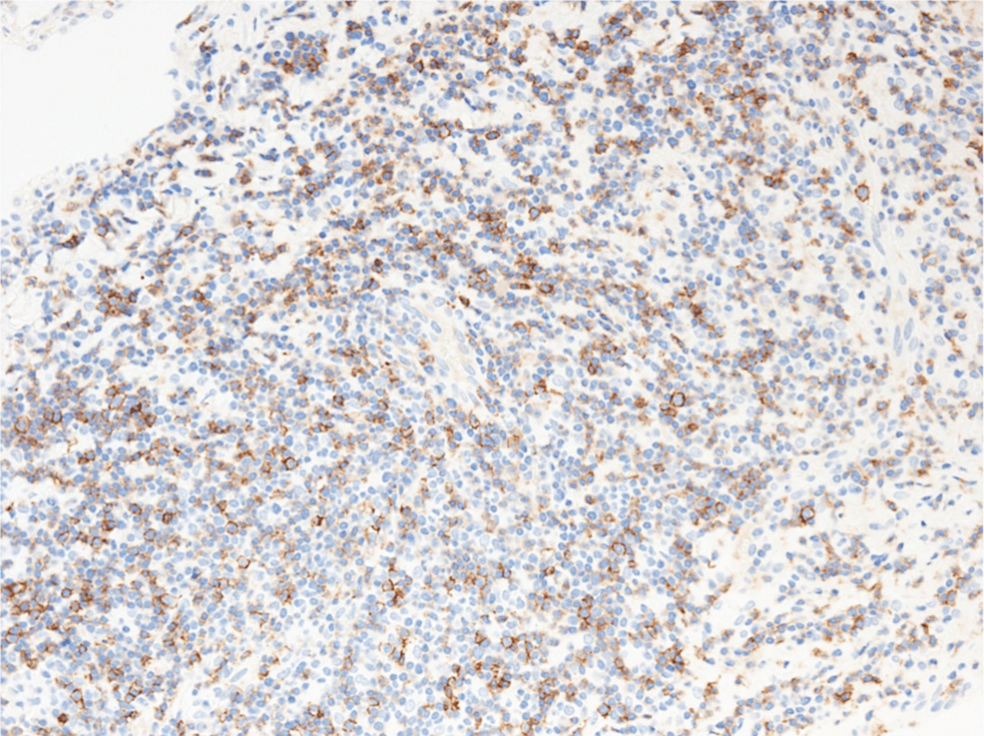
CD56 immunostaining in VATS lung biopsy tissue. Immunostaining in VATS lung biopsy tissue showed medium-sized lymphocytes were partially CD56 positive (original magnification, ×400).

**Figure 8:**
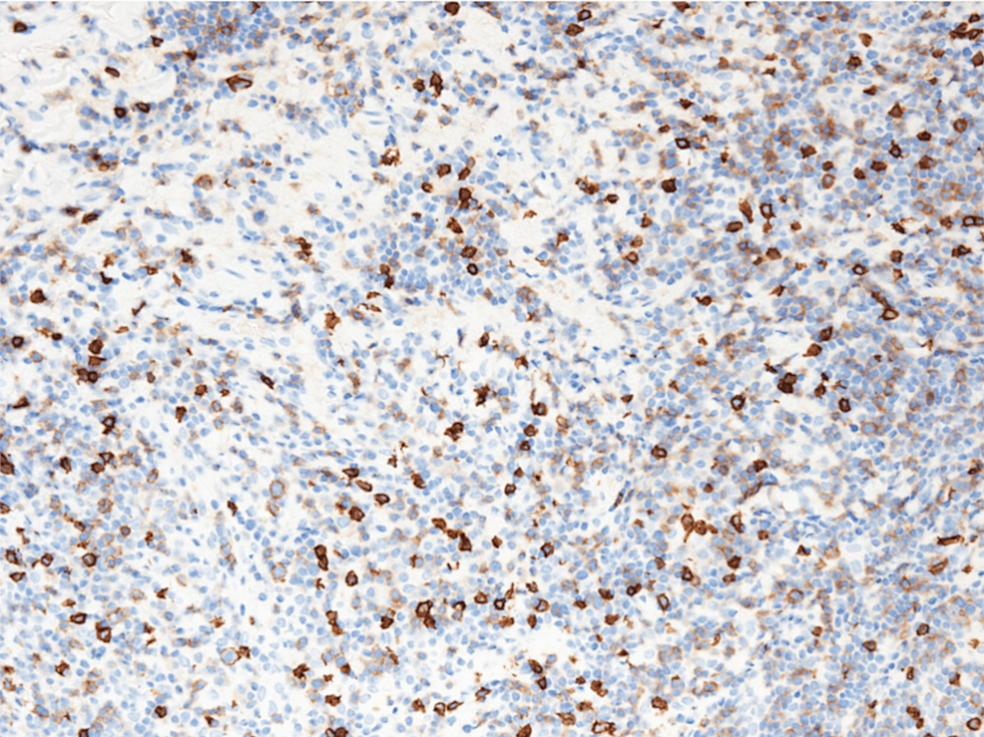
CD8 immunostaining in VATS lung biopsy tissue. Immunostaining in VATS lung biopsy tissue showed medium-sized lymphocytes were partially CD8 positive (original magnification, ×400).

**Figure 9:**
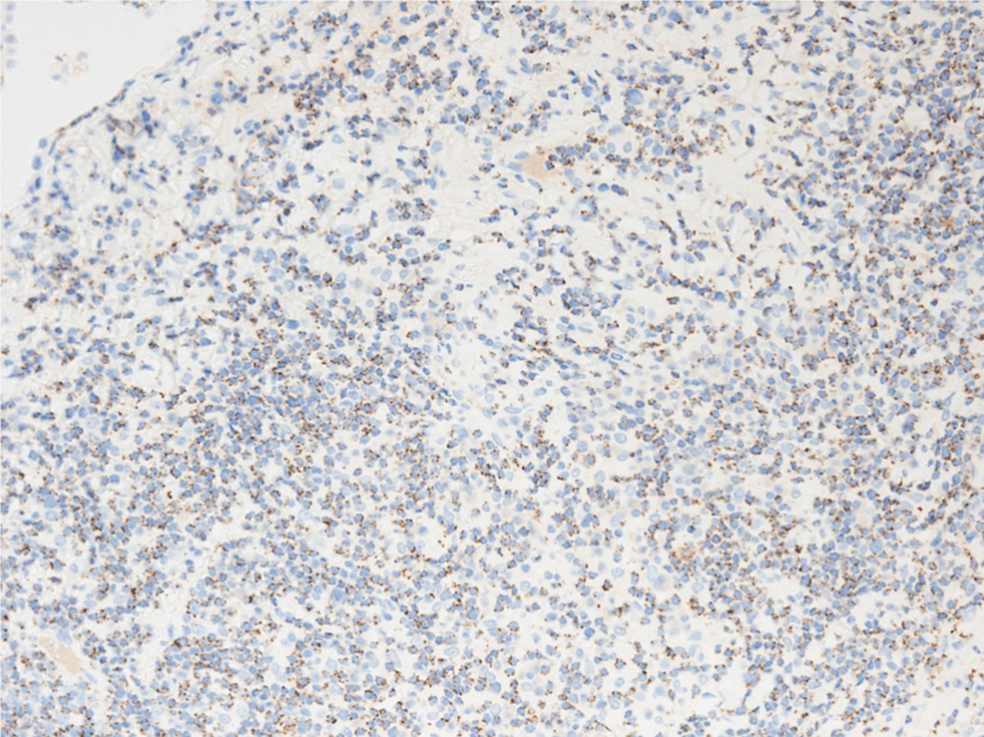
TIA-1 immunostaining in VATS lung biopsy tissue. Immunostaining in VATS lung biopsy tissue showed medium-sized lymphocytes were TIA-1 positive (original magnification, ×400).

**Figure 10:**
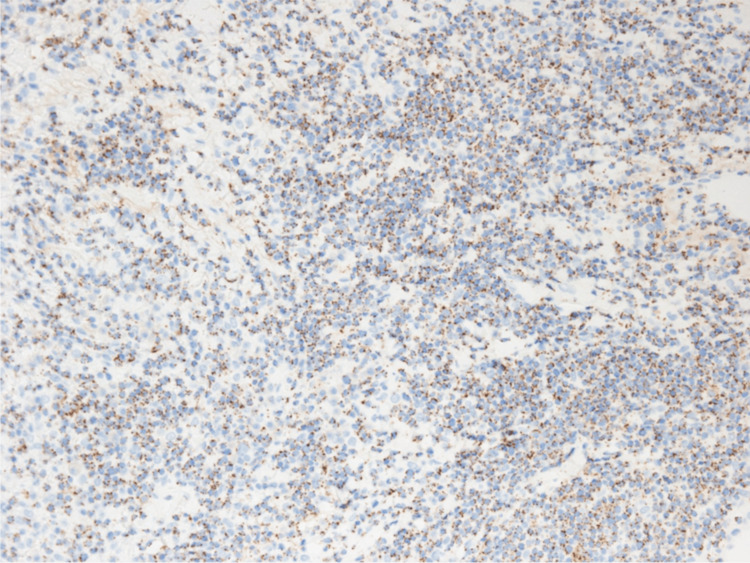
GranzymeB immunostaining in VATS lung biopsy tissue. Immunostaining in VATS lung biopsy tissue showed medium-sized lymphocytes were granzymeB positive (original magnification, ×400).

**Figure 11:**
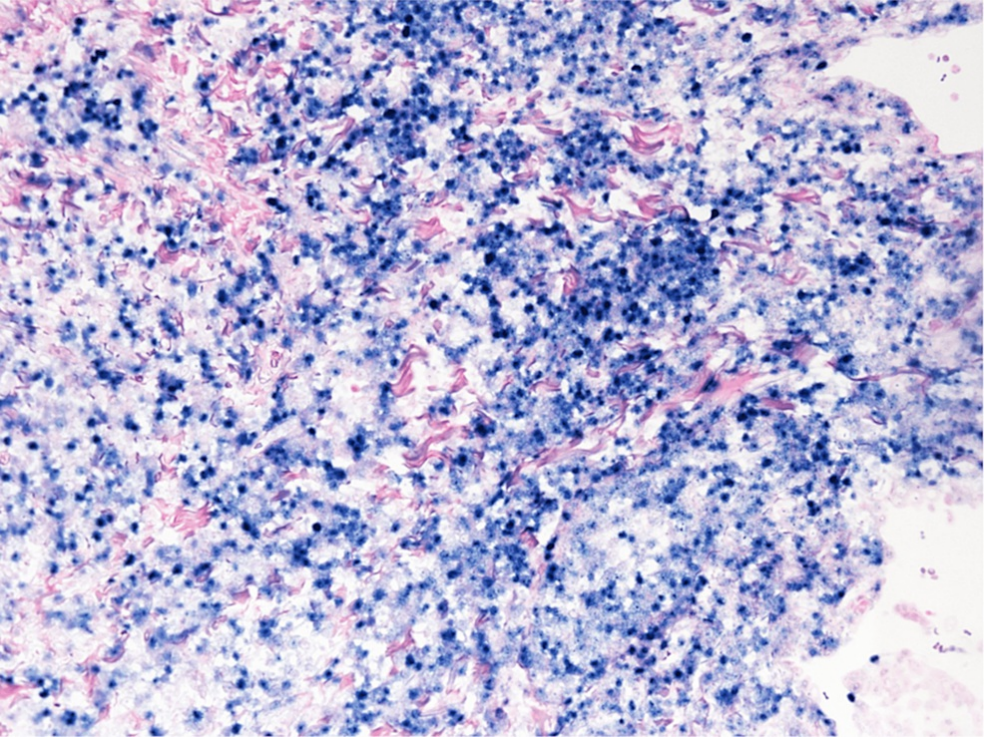
EBV-encoded RNA ISH in VATS lung biopsy tissue. Immunostaining in VATS lung biopsy tissue showed EBV-encoded RNA ISH positive (original magnification, ×400).

Flow cytometry revealed that cells characterized by CD2, CD56 positivity, CD3, CD4, CD5, and CD7 were low in the small cell area. Hematoxylin-eosin staining showed an increase in medium-sized lymphocytes and slightly larger lymphocyte-like cells (Figure 6). Immunostaining showed CD3, CD56 (partial positive) (Figure 7) and CD8 (Figure 8), CD2, T-cell intracellular antigen-1 (TIA-1) (Figure 9), granzyme B (Figure 10) positive, CD20 negative, Epstein-Barr virus (EBV)-encoded RNA in situ hybridization (ISH) positive (Figure 11), poor proliferation (G-band), and no ALK mutated gene (fluorescence ISH). Furthermore, the viral load of EBV-DNA quantified by quantitative polymerase chain reaction using whole blood was 380,000 copies/mL. Resultantly, the patient was diagnosed with primary pulmonary ENKL.

As our hospital did not have a hematologist, the patient was transferred to a high-volume center where a hematologist was available for chemotherapy, which was initiated on day 28 after the VATS lung biopsy. No bone marrow biopsy was performed during our hospitalization. After transfer to a high-volume center, he received chemotherapy with the SMILE regimen (dexamethasone, methotrexate, ifosfamide, l-asparaginase, and etoposide), which showed improvement in his symptoms.

## Discussion

We report a case of primary pulmonary ENKL that was successfully diagnosed by VATS lung biopsy with various symptoms including weight loss and electrolyte abnormalities besides respiratory symptoms. The patient presented with anorexia, weight loss, and multiple symptoms, including multiple infiltrative shadows with diffuse ground-glass opacities in both lung fields, electrolyte abnormalities, and proteinuria, which rendered the diagnosis extremely difficult. From the time of admission, chest CT showed diffuse ground-glass opacities with interlobular septal thickening, suggesting the spread of the lesion through the lymphatic vessels. Histologically diffuse advanced gastric carcinoma, which is sometimes complicated by pulmonary lymphangitis, has been reported [4]. In the present case, upper gastrointestinal endoscopy was performed; however, gastric cancer was not observed. ACE is reportedly elevated in sarcoidosis, and although serum ACE levels are often normal or low in malignant lymphomas, Rømer reported an elevation in one of 27 Hodgkin lymphoma patients [5]. ACE is also reportedly expressed in tumor-associated macrophages in malignant lymphoma tissue [6]. In the present case, large number of macrophages were observed in the BAL; hence, tumor-associated macrophages may have been involved in the elevated serum ACE levels in the BAL (Table 1). Cases of interstitial nephritis due to infiltration of T-cell lymphoma into the kidney have been reported [7]. In the present case, the infiltration of lymphoma cells into the kidneys, including SIADH, may have been involved in the pathogenesis of the disease.

A PubMed search for case reports since 2000 using the keywords “primary pulmonary NK/T-cell lymphoma” yielded 18 case reports written in English (case reports written in Chinese were excluded) (Table 2) [8-15]. To diagnose pulmonary ENKL, eight bronchoscopies were performed in the cases studied, and the diagnosis was made in 50% of them. Fine-needle aspiration is inapplicable for the initial diagnosis of malignant lymphoma because sufficient tissue samples cannot be obtained by this method; hence, incisional or excisional biopsy is preferred [16]. Open lung biopsy or VATS lung biopsy was performed in four cases, with accurate diagnoses in all (Table 2). Furthermore, a comprehensive evaluation by flow cytometry, chromosome, genetic, and immunostaining tests is recommended for diagnosing malignant lymphoma [17]. In the present case, malignant lymphoma was strongly suspected based on the presence of elevated LDH, sIL-2R, and hepatosplenomegaly and the negative results of gastric cancer and sarcoidosis, which were initially suspected as differentials; the case was submitted to ML-NET for these evaluations (Figures 4, 5). Careful preparation, including consultation with a pathologist and a hematologist prior to testing, is necessary when primary pulmonary malignant lymphoma is suspected.

**Table 2:** Summary of reported cases of primary pulmonary NK/T cell lymphoma since 2000 PTN: Percutaneous transthoracic needle, TBB: Transbronchial biopsy, DRC: Deteriorated respiratory condition

No	Age	Gender	Radilogic findings of chest CT	Tests leading to diagnosis	Treatment and outcome	Author	Year
1	72	Female	Consolidation in both lungs	Post mortem examination	DRC, died	P Laohaburanakit [9]	2006
2	34	Female	Lung consolidation in left lower lobe	PTN biopsy	Chemotherapy, died	BH Lee [9]	2006
3	41	Male	Massive infiltrates in both lungs	open lung biopsy	Chemotherapy, died	A Morovic [12]	2010
4	50	Male	Multiple lung nodules in both lungs	post mortem examination	DRC, died	K Oshima [9]	2012
5	73	Female	Soft tissue mass in the right upper lobe	open lung biopsy	DRC, died	L Gong [9]	2013
6	80	Male	Soft tissue mass in the left lower lobe	PTN biopsy	Chemotherapy, died	CH Liu [10]	2014
7	39	Male	Lung consolidation in right lower lobe	TBB	Chemotherapy, alive	W Gui [8]	2015
8	83	Female	Multiple lung nodules in both lungs	CT guided needle biopsy	DRC, died	W Fei [9]	2015
9	46	Male	Lung consolidation in left lower lobe	TBB	DRC, died	S Lee [9]	2016
10	53	Male	Lung consolidation in right lower lobe	TBB	Chemotherapy, died	CC Chien [9]	2016
11	44	Male	Multiple lung nodules in both lungs	2^nd^ TBB	DRC, died	J Zhang [15]	2017
12	55	Male	Diffuse ground glass opacities	VATS	Chemotherapy, alive	M Song [9]	2017
13	34	Female	Multiple nodules with ground-glass opacities	CT guided needle biopsy	DRC, died	Y Qui [9]	2018
14	39	Male	Multiple lung nodules in both lungs	Pleuropuncture, liver biopsy	DRC, died	H Mori [11]	2018
15	20	Male	Multiple lung nodules in both lungs	VATS	Chemotherapy, alive	T Yabushita [14]	2019
16	40	Male	Multiple nodules with ground-glass opacities	CT guided needle biopsy	Chemotherapy, died	Y Wang [13]	2020
17	49	Male	Massive infiltrates in both lungs	CT guided needle biopsy	Chemotherapy, died	Q Hu [9]	2020
18	74	Male	Multiple lung nodules in both lungs	CT guided needle biopsy	Chemotherapy, died	Q Hu [9]	2020

Certainly, in 12 of 18 cases, TBB, PTN, and CT-guided biopsy led to the diagnosis of pulmonary ENKL. However, Mori et al. reported cases in which TBB did not lead to a diagnosis (Table 2). In addition, Oshima et al. and Laohaburanakit et al. reported two cases in which TBB and CT-guided biopsy before death failed to reach the diagnosis, and the diagnosis was made postmortem (Table 2). It is also considered important to obtain a large enough specimen for the diagnosis of malignant lymphoma. Taking these factors into consideration, we believe that VATS lung biopsy should be considered when TBB fails to reach a diagnosis. Bronchoscopic cryobiopsy has not been reported for the diagnosis of primary pulmonary ENKL but has been reported for other pulmonary malignant lymphomas. Bronchoscopic cryobiopsy could be considered at facilities where it is feasible. We have tabulated the advantages and disadvantages of TBB, CT-guided needle biopsy, and VATS lung biopsy in the diagnosis of primary pulmonary malignant lymphoma (Table 3).

**Table 3:** The advantages and disadvantages of TBB, CT-guided needle biopsy, and VATS lung biopsy in the diagnosis of primary pulmonary malignant lymphoma. TBB: Transbronchial biopsy, CT: Computed Tomography, VATS: Video-assisted thoracoscopic surgery, BAL: Bronchoalveolar lavage

Method	Advantages	Disadvantages
TBB	･Minimally invasive ･Direct evaluation of bronchial mucosal lesions is possible. ･Flow cytometric evaluation of BAL may provide information on malignant lymphoma.	･The biopsy tissue obtained may be too small to be evaluated by immunostaining, flow cytometry, or genetic testing. ･Biopsy may be difficult depending on the site and size of the lesion. ･Rarely, complications of biopsy or BAL may cause pneumothorax, hemorrhage, or worsening of respiratory status.
CT guided needle biopsy	･Diagnosis rate may be higher than bronchoscopy in the case of peripheral lung lesions.	･The biopsy tissue obtained may be too small to be evaluated by immunostaining, flow cytometry, or genetic testing. ･The risk of complications from pneumothorax is high. ･The facilities that can perform the procedure are limited by equipment and personnel.
VATS lung biopsy	･Direct observation of the lesion is possible. ･The biopsy tissue obtained is large. ･Diagnosis rate is very high.	･General anesthesia is required, and there is a risk of perioperative complications such as worsening of respiratory condition. ･Patients in poor respiratory condition may have difficulty undergoing the procedure. ･Facilities that can perform the procedure are limited by equipment and personnel.

In the present case, weight loss was considered a systemic B symptom. A prognostic model predicting the clinical outcome of ENKL showed that age >60 years, Ann Arbor classification stage III or IV, distant lymph node metastasis, non-nasal type disease, and detectable titer of viral DNA were significantly associated with an overall three-year survival [18]. The three-year overall survival rates for low-risk (zero/one risk factor), intermediate-risk (two risk factors), and high-risk (three or more risk factors) patients were reportedly 81%, 55%, and 28%, respectively. This patient was considered to have stage IV disease because of the diffuse spread of the disease to the lungs. Moreover, the patient had three risk factors, including non-nasal lesions and detectable titers of viral DNA, and was considered to be in a high-risk group. Additionally, the patient's chest imaging findings worsened over time (Figure 1), making the decision for highly invasive testing in this case clinically difficult.

## Conclusions

In conclusion, we report a case of primary pulmonary ENKL, successfully diagnosed by VATS lung biopsy. This case demonstrates that VATS lung biopsy and flow cytometry, chromosomal, genetic, and immunostaining tests are one of useful diagnostic tools when primary pulmonary malignant lymphoma is suspected.
